# Attenuation by l-thyroxine of oxidant-induced gut epithelial damage 

**DOI:** 10.22038/ijbms.2019.37169.8852

**Published:** 2019-09

**Authors:** Zahra Shahedi, Masoumeh Varedi, Zohreh Bagheri, Afagh Moatari, Hengameh Sharafpour

**Affiliations:** 1 Department of Physiology, School of Medicine, Shiraz University of Medical Sciences, Shiraz, Iran; 2 Department of Microbiology & Virology, School of Medicine, Shiraz University of Medical Sciences, Shiraz, Iran

**Keywords:** Epithelial barriers, Gut, Oxidative stress, Thyroid hormones, Trauma

## Abstract

**Objective(s):**

Severe injuries are often associated with tissue hypothyroidism, elevated damaging mediators in circulation, and broken gut epithelial barrier. However, the relationships between the hypothyroid state and gut epithelial damage are largely unknown. Therefore, in this study, we investigated the effects of L-thyroxine (T4) on *in vitro* models of intact and compromised gut epithelium.

**Materials and Methods:**

Gut epithelium equivalent was generated by cultivation of IEC-18 rat intestinal epithelial cells into transwell inserts. Confluent cultures were then compromised by scratching or H_2_O_2 _and traumatized rat sera (TUR sera) treatments. Macromolecules permeation and transepithelial electrical resistance (TEER) were evaluated by conventional methods. Morphology and scratch wound closure were assessed microscopically. Cell viability/proliferation was assessed by MTT assay.

**Results:**

Both H_2_O_2_ and TUR sera induced marked yet different types of epithelial disintegration. While H_2_O_2_ significantly increased and decreased probe permeation and TEER, respectively, TUR sera was ineffective. Cultures treated with normal rat sera (sham sera) exhibited morphology, probe permeation, and TEER comparable to those of control cultures. Presence of T4 attenuated the H_2_O_2_-induced but not TUR sera-induced damages. T4 treatment accelerated, albeit marginally, wound closure but had virtually no effects on cell viability/proliferation.

**Conclusion:**

These data suggest that different mechanisms are involved in oxidant- and trauma-induced gut epithelial barrier breakdown. Besides, they show that T4 markedly attenuates oxidant-induced gut epithelial damage. Accordingly, one may also conclude that tissue hypothyroidism does not contribute to trauma-induced gut barrier breakdown.

## Introduction

Trauma and severe illnesses may cause multiple system organ failure (MSOF), a lethal condition that occurs due to epithelial/ endothelial barriers breakdown (1-3). Although the exact mechanisms involved in the trauma-induced epithelial damages are not well known, available data indicate that oxidants and harmful mediators such as inflammatory cytokines are involved (4-6). Among barriers, the gut seems to play a critical role. Broken gut epithelium allows bacterial translocation that may boost the mediators and accelerates MSOF development (3, 6, 7). We have previously shown that intestinal epithelium is severely damaged in scald injury rats (8, 9). Besides, we found that scald injury rat sera disintegrate monolayer culture, inhibit migration and proliferation, and induce apoptosis in rat intestinal epithelial cells (8, 9). Our data, along with those reported by other researchers, fortify the contribution of circulating mediators to the trauma-induced gut epithelial damages. 

In addition to barriers breakdown, tissue hypothyroidism and reduced levels of thyroid hormone (TH) are reported in trauma and critically ill patients (10-14). Normal level of TH is essential for cellular homeostasis (15, 16) and, hence, alterations in TH level may disturb the normal operation of multiple organs, including gut epithelium. Cellular intactness, solute transporters, and tight junctions, elements that cooperatively determine permeability weight, are the major role players in the function of an epithelial barrier (17-20). It is now accepted that a junctions–associated contractile apparatus may modulate epithelial permeability through the paracellular pathway (17, 19, 21, 22). Previous studies have shown that modulation by TH of epithelial functions is mediated not only through the expression of water channels and solute transporters but also through junctions-associated contractile proteins (23-27). In this regard, we have previously shown that hypothyroidal state promotes and hyperthyroidal state, in contrast, inhibits trauma-induced alveolar barrier hyperpermeability through junctions-associated contractile activity (23, 28). In addition, TH is shown to be involved in the renovation and maintenance of epithelial barriers; the gut in particular (29, 30). According to these findings, one may conclude that tissue hypothyroidism may promote gut epithelial breakdown in trauma and critically ill patients. Therefore, the aim of this study was to examine the effects of L-thyroxine (T4) on the compromised gut epithelial barrier. 

## Materials and Methods

***Materials***

Tissue culture media and reagents were purchased from Gibco or Shellmax companies. Transwell inserts were purchased from Corning Company (Germany). IEC-18 rat intestinal epithelial cells were obtained from Pasture Institute (Tehran, Iran). Tissue culture-tested L-thyroxin, hydrogen peroxide, FITC-conjugated dextran-4 (FD-4) probe, and all other reagents were purchased from Sigma (Steinheim, Germany). 

***Methods***

*Animals, trauma induction, and sera collection*

Adult male Sprague-Dawley rats weighing 270–300 g were obtained from laboratory animal house, Shiraz University of Medical Sciences, Shiraz, Iran. Animals had free access to commercial rat chow and water. The room temperature (22 ± 2 ^°^C) and lighting (on from 7:00 am to 7:00 pm) were monitored daily. Animals were traumatized by high tidal volume mechanical ventilation, as described previously (23). In brief, animals were anesthetized and subjected to tracheostomy. Then, the animals were either connected to a volume-controlled ventilator (traumatized rats) or left to breathe normally (sham rats). By the end of the ventilation period, heart blood samples were collected from traumatized and sham animals. Sera were then harvested and stored at -20 ^°^C until used for tissue culture experiments. Animal Care and Use Committee of Shiraz University of Medical Sciences approved the experimental protocols. 

*Cells, epithelial barrier construction, and transepithelial electrical resistance assessment*

IEC-18 rat intestinal epithelial cells were grown and passaged according to the protocol previously described for IEC-6 cells (8). Gut epithelial barrier equivalent was generated by seeding the cells into transwell inserts (31). In brief, 5x10^4^ cells in 500 µl culture media were seeded into transwell inserts, holding permeable membrane, and grown for at least three days. From day three on, transepithelial electrical resistance (TEER) was measured using EndOhm and EVOM2 apparatus (World Precision Instruments, USA). Cultures with constant TEER for three consecutive days were used for experiments. Protocols provided by the manufacturer were followed during TEER assessment and system calibration. When morphology of the epithelium was to be tested, cells were seeded into inserts holding polyester membrane because it confers better optical clarity and cell visibility as compared to polycarbonate membrane. 

*Epithelial injury induction and permeability assessment*

Epithelial injury was induced by exposing confluent cultures to H_2_O_2_ for 30 min (1 mM, final concentration) or TUR sera (5%) for 32 hr. Sham and control cultures were exposed to normal rat sera (sham sera) and FBS, respectively. By the end of the incubation periods, FD-4 probe was added to the apical side of the inserts (1 mg/ml final concentration). Media samples were then collected from the basal side of the inserts at 30 min interval for 2 hr. The fluorescent activity of the samples was immediately measured by spectroscopy (excitation and emission of 487 nm and 518 nm, respectively). The readings were then applied to the corresponding standard curves, and the concentration of FD-4 was determined accordingly. When the effects of T4 were to be tested, cells were exposed to a physiologic (0.1 μ M) or supraphysiologic (5 μ M) dose of T4, 2 hr prior to H_2_O_2_ or TUR sera treatment. The effects of T4 on a different type of epithelial injury, scratch wound, were also examined. The wounds were generated according to a previously described protocol (8). Briefly, cells were seeded into 6-well plates and grown in regular media. On the day of experiment, one wound/well was generated in confluent cultures. The cultures were then washed with PBS, and the medium was replaced by fresh media supplemented with 1% FBS with or without T4. Wound area was recorded immediately and considered as original wound size. Wound closure, as an index of cell motility, was monitored for 6–8 hr, and recorded by photography. Photomicrographs were then digitalized by the aid of ImageJ software. 

*Cell viability assessment*

Cell viability/proliferation was estimated by conventional MTT assay. In brief, cells (7x10^3^) were seeded into a 96-well plate and grown in regular culture media. On the day of the experiment, semi-confluent cultures were treated with fresh media supplemented with 1% FBS with or without T4 (5 µM final concentration) and incubated for 48 hr. The MTT solution was then added to the culture media, and the plate was incubated for 4 hr to let the conversion of yellow MTT to purple formazan crystals. At the end of this incubation period, formation of formazan crystals was examined microscopically. To dissolve the crystals, culture media was aspirated, and an equal volume of DMSO was added to each well precisely. The reactions were then quantified by colorimetric method using a BioTech plate reader. 

***Statistical analysis***

 The data were analyzed by the aid of Statistical Package for the Social Sciences (SPSS, ver. 23). Significant differences between test groups were determined by one-way analysis of variance followed by the Mann-Whitney test. The paired Student t-test was used for probe permeation data analysis. For all statistical analysis, a *P*-value<0.05 was preset as the level of significance. 

## Results

***Effects of H***_2_ ***O***_2_ ***and TUR sera on epithelial morphology in the presence and absence of T4***

**Figure 1:**
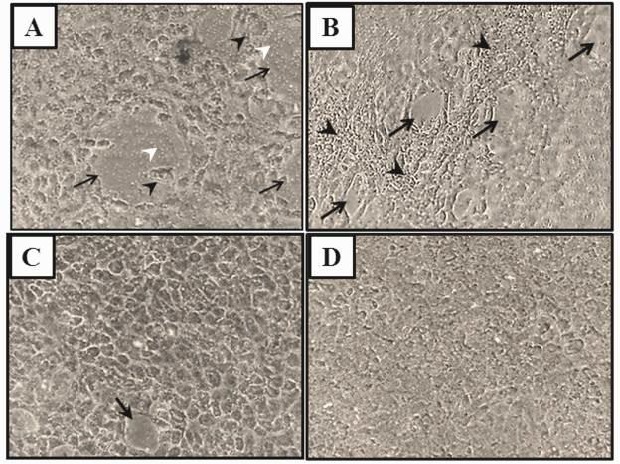
Representative photomicrographs show* in vitro*-made gut epithelium treated with H_2_O_2_ (A) or traumatized rat serum, TURsera, (B). Both treatments induced epithelial damages (black arrows) that were different in nature. Presence of T4 attenuated the H_2_O_2_-induced (C) but not TURsera-induced (not shown) damages. The epithelium treated with normal rat sera, sham sera, (D) or FBS (Control, not shown) remained intact. Black arrowheads show cell necrosis and compaction in the H_2_O_2_-treated and TUR-treated epithelia, respectively. White arrowheads point to the transwell membrane pores that are exposed at the injury sites

As shown in Figure 1, cultures treated with H_2_O_2_ showed rapid and progressive epithelial disintegration. Within one hour, multiple large wounds with rough margins appeared throughout the culture (Figure 1A). Cells located at the wound edges exhibited severe cell swelling, characteristic of necrotic cells. Besides, many cells in the culture exhibited arborized plasma membrane indicating that cells are not in a normal state. There were no indications of cell retraction or compaction around the wounds. Although with a rather long delay of 32 hr, TUR sera induced epithelial disintegration as well. From morphological point of view, however, the wounds were very different from those induced by H_2_O_2_. They were smaller and exhibited smooth margins. No necrotic or apoptotic cells were detected. Interestingly, a contracture-like reaction was detected in close proximity of almost all wounds (Figure 1B). Pretreatment with T4 attenuated the H_2_O_2_-induced (Figure 1C) but not TUR sera-induced (data not shown) epithelial disintegration. Morphological feature of the sham sera-treated cultures was rather similar to that of (Figure 1D) control cultures. However, sham sera-treated cultures exhibited multilayer rather than monolayer structure at a few spots, suggesting that growth-promoting effects of homologous sera are greater than those of heterologous sera.

***Permeability and TEER of the compromised epithelium in the presence and absence of T4***

**Figure 2:**
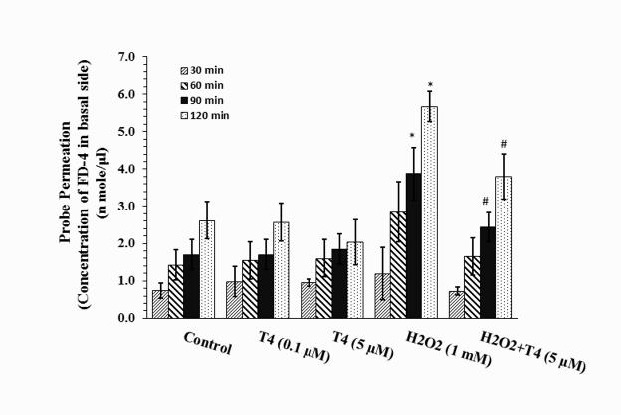
Fluorescein isothiocyanate dextran-4 KD (FD-4) probe permeation measure of intact (Control) and H_2_O_2_-compromised gut epithelium in the presence or absence of physiologic (0.1 μM) and supraphysiologic (5 μM) doses of T4. Probe permeation was measured for 2 hr at 30 min interval. Each bar shows Mean±SEM of the data collected from 4 to 5 individual experiments. *P<0.05, significant differences in comparison with corresponding time points of control culture. #*P*<0.05, significant differences in comparison with corresponding time points of H_2_O_2_-treated cultures (H_2_O_2_)

**Figure 3:**
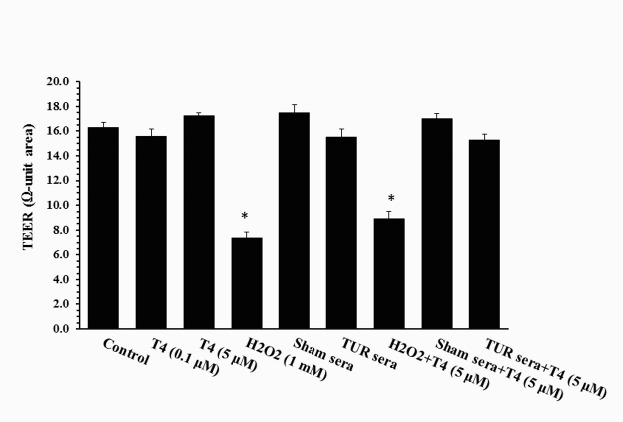
Transepithelial electrical resistance (TEER) of gut epithelium treated with H_2_O_2_, traumatized rat sera (TURsera) or normal rat sera (Sham sera) in the presence and absence of physiologic (0.1 μM) and supraphysiologic (5 μM) doses of T4. Control cultures were treated with FBS. Each bar presents mean±SEM of 4-5 individually tested epithelia. *P<0.05, significant differences in comparison with control and H_2_O_2_-treated epithelia

Probe permeations of the cultures are shown in Figure 2. As the bar graphs show, control cultures were somewhat permeable to FD-4 probe. Permeability of H_2_O_2_-treated cultures was greater than that of control cultures. Statistical analysis revealed that the differences are significant (*P*≤0.05) at 90 min and 120 min, but not 30 min and 60 min after probing. Concentrations of the probe in samples collected from H_2_O_2_-treated cultures were 3.88±0.7 n mole/μl and 5.68±0.3 n mole/μl at 90 min and 120 min time points, respectively, which are significantly higher than those of control cultures at the corresponding time points (1.70±0.3 n mole/μl and 2.63±0.4 n mole/μl at the indicated time points, respectively). Neither physiologic (0.1 µM) nor supraphysiologic (5.0 µM) dose of T4 had significant effects on the permeability of control cultures. However, the H_2_O_2_-mediated hyperpermeability was significantly (*P*≤0.05) reduced in the presence of T4 (2.58±0.3 n mole/μl, 3.80±0.3 n mole/μl). Statistical analysis revealed that the reducing effects of T4 are significant (*P*<0.05). The H_2_O_2_-induced hyperpermeability was associated with a significant reduction in TEER. As shown in Figure 3, H_2_O_2_ caused a significant drop in TEER (7.0±0.5 Ω-unit area vs 17±0.9 Ω-unit area; *P*≤0.05). Although pretreatment with T4 significantly attenuated the H_2_O_2_-induced hyperpermeability, it did improve TEER marginally. Likewise, T4 had no significant effects on TEER of control cultures. Control cultures exhibited similar TEER in the presence and absence of the physiologic and supraphysiologic doses of T4 (17±0.6 Ω-unit area and 17±0.3 Ω-unit area, respectively).

Cultures treated with TUR sera and sham sera showed reduced probe permeation when compared to that of the control culture. However, statistical analysis revealed that the reductions are not significant. Pretreatment with T4 did not alter probe permeation in these cultures. Likewise, neither TUR nor sham sera had significant effects on TEER (Figure 3). 

**Figure 4:**
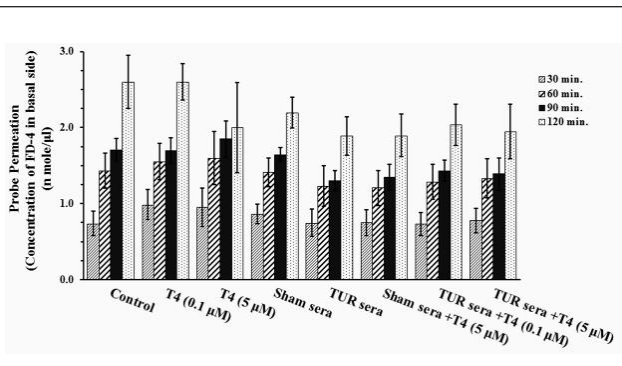
Fluorescein isot hiocyanate dextran-4 KD (FD -4) probe permeation measure of gut epithelium treated with traumatized rat sera (TURsera) or normal rat sera (Sham sera) in the presence or absence of physiologic (0.1 μM) and supraphysiologic (5 μM) doses of T4. Control cultures were treated with FBS. Probe permeation was measured for 2 hr at 30 min intervals. Each bar presents mean±SEM of 4-5 separate experiments

**Figure 5:**
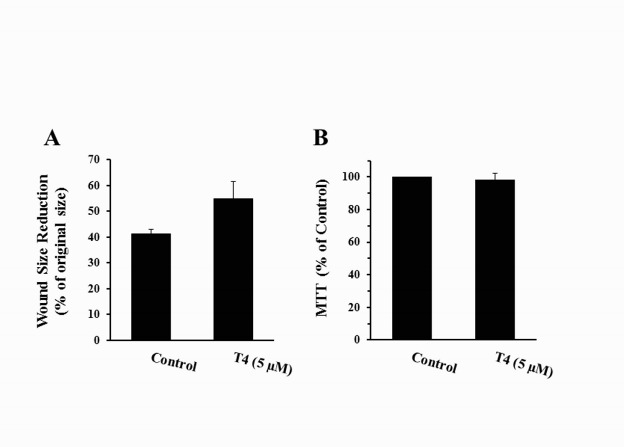
Effects of supraphysiologic dose of T4 on viability (A) and motility (B) of IEC-18 cells evaluated by MTT and scratch wound healing assays, respectively. Each bar shows mean±SEM of 4 (MTT assay) or 3 (Wound closure assay) separate experiments. At least, 12 wounds were examined in each experiment

***Epithelial cell mobility and viability in the presence and absence of T4***

To determine whether attenuation by T4 of H2O2-induced epithelial disintegration is mediated through activation of cell motility and/or viability, the rates of scratch wound closure and cell proliferation were assessed in the presence and absence of T4. As shown in Figure 5A, wound size reduction was greater in T4-treated cultures as compared to those in control cultures, indicating that T4 may accelerate IEC-18 cells motility and promote epithelial resealing. However, statistical analysis of the data revealed that T4-mediated cell motility is marginally significant (*P*≤0.06). Cell proliferation in cultures treated with a supraphysiologic dose of T4 was comparable to those of control cultures, suggesting that proliferation of IEC-18 cells is not affected by T4 (Figure 5B). 

## Discussion

The data of the present study suggest that H_2_O_2_ compromises gut epithelium and that T4 attenuates the damage, at least in part, through inhibition of cell death. Morphological alterations such as cell swelling and plasma membrane blebbing, in conjunction with the absence of epithelial contracture, in the H_2_O_2_-treated cultures indicate that epithelial disintegration and hyperpermeability are mostly mediated through cell death; necrosis perhaps. The cytotoxic effects of H_2_O_2_ reported here are in agreement with previous findings (32-34). Although more recent studies suggest that cytotoxic effect of H_2_O_2_ is mediated through multiple pathways (11, 35-38), early studies show that it is mainly mediated by increased lipid peroxidation and plasma membrane disintegration (32, 39); typical events in the process of cell necrosis. 

Thyroid hormones play important roles in development, maintenance, and renovation of the gut epithelium (29, 40-42). Our results show that the H_2_O_2_-induced epithelial disintegration and hyperpermeability are significantly inhibited in the presence of T4. We assumed that T4 might have mediated the effects by activation of cell growth and/or motility. Despite marginal activation by T4 of cell motility, the results of MTT and the insignificant effect of T4 on cell motility ruled out the assumption, and suggested to us that the improving effects of T4 are mediated largely through inhibition of cell death. 

As originally described by Quaroni and May, IEC-18 cells are rat intestinal crypt cells and exhibit many crypt cell criteria including higher proliferation rate and more leaky junctions as compared to differentiated villus epithelial cells (43). In fact, both transcellular and paracellular pathways contribute to the permeability weight of a barrier. While the former depends on solute transporter proteins and water channels, the latter depends on cell junctions. Accordingly, the epithelial hyperpermeability induced by H_2_O_2_ may have been mediated not only through cell death but also through modulation of transporters and intercellular junctions. Data reported by Kevil and colleagues support the hypothesis. Using *in vitro* model of endothelial barrier, they have shown that, H_2_O_2_-induced hyperpermeability is associated with alterations in junctional proteins and associated kinase/phosphatase activities (44, 45). There is evidence to show that T4 potently inhibits several kinases, including those involved in regulation of paracellular pathway (46). We acknowledge that the present study does not address the exact underlying mechanisms of attenuation by T4 of the H_2_O_2_-induced epithelial disintegration. However, we have previously shown that supraphysiologic levels of TH improve alveolar fluid clearance by a reduction in phosphomyosin light chain, a cell junction-associated protein, and up-regulation of aquaporin five protein expression (23). Whether attenuations by T4 of H_2_O_2_-induced effects are mediated, at least in part, through the same mechanisms, remain to be determined. Furthermore, T4 may bind to its cell surface receptor, αvβ3 integrin, and activates different cell motility- and survival-related signaling pathways that may have contributed to the T4 effects (47). 

We have previously shown that sera collected from burn injury rats compromise gut epithelium, disintegrate monolayer culture, and alter gene expression of rat intestinal epithelial IEC-6 cells (8-10). Although in the present study, the nature of injury, cell culture model, and the cell type are different, TUR sera exerted somewhat similar effects on the cells. Surprisingly, TUR sera disintegrated the cell monolayer culture but had no effects on probe permeation measure and TEER. Given that TUR disintegrated the epithelium through contracture rather than cell death, one possible explanation for the unchanged probe permeation and TEER in the TUR sera-treated cultures is that increased probe permeation in the disintegrated regions is counterbalanced by reduced probe permeation in the compacted area. 

## Conclusion

The results of the present study suggest that trauma-mediated damaging factors and oxidative stress compromise intestinal epithelial barrier through different mechanisms and that healing effects of T4 on epithelial damage depend on the type of injury. Since the mechanism of epithelial resealing depends on developmental stage, species, tissue type, and nature of the damage, we believe that consumptive hypothyroidism may not contribute to all forms of trauma-induced barriers dysfunction. 
